# The Efficacy of Using Combination Therapy against Multi-Drug and Extensively Drug-Resistant *Pseudomonas aeruginosa* in Clinical Settings

**DOI:** 10.3390/antibiotics11030323

**Published:** 2022-02-28

**Authors:** Frank Jones, Yanmin Hu, Anthony Coates

**Affiliations:** Institute for Infection and Immunity, St George’s University of London, London SW17 0RE, UK; m1801022@sgul.ac.uk (F.J.); ymhu@sgul.ac.uk (Y.H.)

**Keywords:** *Pseudomonas aeruginosa*, combination therapy, extensively drug resistant (XDR), multidrug resistant (MDR), carbapenems, beta-lactams, ESBLs

## Abstract

*Pseudomonas aeruginosa* is a Gram-negative bacterium which is capable of developing a high level of antibiotic resistance. It has been placed on the WHO’s critical priority pathogen list and it is commonly found in ventilator-associated pneumonia infections, blood stream infections and other largely hospital-acquired illnesses. These infections are difficult to effectively treat due to their increasing antibiotic resistance and as such patients are often treated with antibiotic combination regimens. Methods: We conducted a systematic search with screening criteria using the Ovid search engine and the Embase, Ovid Medline, and APA PsycInfo databases. Results: It was found that in many cases the combination therapies were able to match or outperform the monotherapies and none performed noticeably worse than the monotherapies. However, the clinical studies were mostly small, only a few were prospective randomized clinical trials and statistical significance was lacking. Conclusions: It was concluded that combination therapies have a place in the treatment of these highly resistant bacteria and, in some cases, there is some evidence to suggest that they provide a more effective treatment than monotherapies.

## 1. Introduction

*Pseudomonas aeruginosa* is a Gram-negative bacterium in the family of *Pseudomonadaceae* [1]. As an opportunistic pathogen, it can readily adapt to its environment through the use of its multiple metabolic pathways and intrinsic antibiotic resistance; this allows it to often survive in medical equipment, water systems, ventilators, and even in some disinfectants [2]. *P. aeruginosa* currently represents about 10% of all hospital-acquired infections worldwide and, as a result of its aforementioned adaptability, it has become a therapeutic challenge due to its high levels of resistance to nearly all of the known monotherapy antibiotics [3,4].

Although *P. aeruginosa* is often resistant to many different antibiotics, if it is carbapenem resistant this is most problematic for the patients. The mechanisms of resistance to carbapenems include the production of β-lactamases, efflux pumps, and mutations that alter the expression or function of the porins and penicillin-binding proteins [5]. However, it is the combination of many of these mechanisms together with resistance to other antibiotics that leads to the high levels of resistance which are seen in some strains of *P. aeruginosa*.

The most common form of resistance to carbapenems comes from the control of cell permeability through the porins, more specifically the control of the permeability to antibiotics entering the cell. With regards to carbapenems, this is due to the alteration or decreased expression of the outer membrane porin OprD [6]. Beta-lactamase-producing *Pseudomonas* strains are also seen, which cause β-lactamase resistance [7]. Of the *P. aeruginosa* strains that do not produce β-lactamases, the loss of OprD functionality is a major form of high-level resistance to β-lactams [8].

Efflux pumps play a key part in the antibiotic resistance that is found in *P. aeruginosa.* These work by secreting small molecules out of the cell system [9] and each pump is specific to a given molecule. There are 12 known efflux pumps, of which 4 work specifically against fluoroquinolones (such as ciprofloxacin, which is commonly used in combinations) [10]. However, it is the overexpression of any specific type of efflux pump which will produce a noticeable decrease in the bacterium’s susceptibility to some antibiotics [11]. The overuse of some drugs will even cause the upregulation of multidrug efflux pumps, such as the MexXY-OprM efflux pump, which when overexpressed leads to reduced susceptibility to aminoglycosides, β-lactams, and fluoroquinolones [12].

The increasing trend towards a more resistant *P. aeruginosa* is brought about by the continued overuse of various antibiotics. For example, carbapenems, which subject the *P. aeruginosa* population to an antibiotic selection pressure. This selection pressure would eventually lead to a more resistant population [1,13]. Speaking more specifically, these more resistant populations are a consequence of a dramatic mutational event in the core genetics of the bacterium, or they can be obtained through horizontal gene transfer [14], therefore resulting in the overexpression of efflux pumps, porins, and endogenous β -lactamases.

Much of *P. aeruginosa*’s resistance stems from the accessory genome, which is a sequence of genes containing integrons, transposons, insertion sequences, genomic islands, prophages, and plasmids [15]; these accessory genomes are mostly strain-specific and generally encode for the adaptations that are acquired within that strain, many of which will be adaptations leading to antibiotic resistance [14,16].

Globally, intensive care units (ICUs) represent a core source of the development and amplification of these antibiotic-resistant bacteria [17,18]. This is due to the frequent use of antibiotic treatments in ICUs and, in addition, the reduced immune responses of the patients who are admitted (likely due to other illnesses), which puts them at a higher risk of harbouring and developing an infection from these bacteria. The risk is further increased when undertaking invasive procedures such as intubation and catheterization [17]. In short, this creates a high selection pressure which allows for the emergence of highly drug-resistant pathogens, such as *P. aeruginosa* [18].

For humans, the issue of antibiotic resistance has existed since shortly after the inception of the antibiotic; however, it is an ever-growing problem that poses a real threat in the future if it is not dealt with. We are seeing very few bacterial strains showing critically high resistance [19,20]; however, it is where these bacteria flourish which makes them such a key issue when they are resistant. While *P. aeruginosa* can be found in the normal intestinal flora, it can also inhabit medical equipment, namely in the ICU [2,17,18]. It has been found that critically ill patients in the ICU have contracted a wide range of nosocomial *P. aeruginosa* infections, such as ventilator-associated pneumonia, gastrointestinal infections, urinary tract infections, and various forms of sepsis [21]. These ICUs represent a core source of the development and amplification of these antibiotic-resistant bacteria [17,18].

Since the resistance of *P. aeruginosa* to monotherapy carbapenems is now widespread [22], the combination of drugs is one way to increase the efficacy of a treatment. For example (Table 1), the combination of a beta-lactam, such as a carbapenem, with a drug which neutralizes the bacterial beta-lactamase results in a greater antimicrobial effect [11]. Some of these combinations have already seen approval by the Food and Drug Administration (FDA), such as ceftazidime–avibactam and ceftolozane–tazobactam. Others use a different approach, such as imipenem–cilastatin, within which the cilastatin inhibits renal dehydropeptidase which destroys imipenem. This combination, including the addition of a beta-lactamase inhibitor called relebactam, has been approved by the FDA. A monotherapy drug called cefiderocol is still in development [23]. During the past six years, a serious problem with new antibiotics which reach the market has arisen, namely that companies have had insufficient sales to provide a reasonable return to their shareholders. The only market success is ceftazidime–avibactam, which was approved by the FDA in 2015 and continues to sell well. During this period, many companies have failed to thrive and some have disappeared. A further prominent issue with clinical authorisation is its slow speed. It has been reported that some strains begin to show resistance to new antibiotics before their FDA approval [24].

In clinical practice, there is already a strong precedent for using combinations to broaden the effective antimicrobial spectrum, often despite the lack of FDA or EMA approvals [25,26]. This includes the use of combinations in order to extend the spectrum of an antibiotic to bacteria which have become resistant to one of the antibiotics in the regimen. An example of this is the approved combination of ceftazidime–avibactam, which is active against bacterial beta-lactamases that destroy ceftazidime [27]. The avibactam in the combination inhibits Ambler classes A, C and some D beta-lactamases.

There is debate, however, regarding whether combination therapies reduce the emergence of resistance in bacteria. This is largely due to the lack of large-scale randomised clinical trials. The relatively low numbers of resistant *P. aeruginosa* infections and the very high costs of the trials mean that it is unlikely that these data will become available in the foreseeable future.

We conducted a systematic search with screening criteria using the Ovid search engine and the Embase, Ovid Medline, and APA PsycInfo databases in order to study the efficacy of using combination therapy to treat multi-drug and extensively drug-resistant *P. aeruginosa* infections in clinical settings.

## 2. Results and Discussion

In a 104-patient study (Table 2 and Table 3), Crusio et al. [28] investigated the clinical success of a polymyxin B combination therapy for the treatment of carbapenem-resistant Gram-negative bacteria. All of the *P. aeruginosa* infections that were included in the study were resistant to penicillins, cephalosporins, quinolones, macrolides, tetracyclines, aminoglycosides (gentamicin and amikacin), and carbapenems (imipenem and meropenem). It was found that of the 11 patients (10.5% of the study’s cohort) who received the combination therapy, two experienced a microbiological cure, but half had died after 6 months. The combinations that were used were one of the following: polymyxin B with a carbapenem, polymyxin B with a carbapenem and rifampin, polymyxin B with ampicillin–sulbactam, or polymyxin B with a carbapenem and tigecycline. These results may indicate that there is little benefit that can be derived from combination therapies, despite the treatment of patients who were infected with bacteria that were completely susceptible to polymyxin B. However, the lack of comparator monotherapies and statistical significance due to the small sample size means that this study does not prove the benefit or disbenefit of combinations.

In the cohort study that was presented by Rigatto et al. [29], 101 patients (Table 2 and Table 3) were included with an average age of 65.2 years. There were 18 cases of drug-resistant *P. aeruginosa* infections in the cohort. Of these 18 cases, the treatment was split between a polymyxin B monotherapy and a combination therapy; 3 of the patients (9.1%) received the combination therapy and 15 (22%) were treated with the monotherapy. The combination that was used was polymyxin B with “an antimicrobial [that was] lacking in vitro activity”, meaning an antibiotic to which the sample strains were resistant. None of the patients who received the combination therapy died, whereas 14 (93.4%) of the patients who received the monotherapy died, this is despite polymyxin B generally showing no resistance (determined at <2 mg/L) in *P. aeruginosa* populations. In contrast to the study by Crusio et al., these results indicate a possible link between the use of combination therapy and lower mortality rates when directly compared to monotherapies. Once again, the sample sizes are small compared to the total study population, so these findings would not be considered statistically significant, but the large disparity between the 93.4% mortality rate with monotherapies and the 0% rate with combination therapies is worthy of note. In this study it was also found that, while all of the strains that were tested were susceptible to polymyxin B, the bacteria were resistant to other antimicrobials that were used in the combination. In spite of this, the combination was shown to perform better than the single polymyxin B therapy. There is little mention of synergy in the paper, but it is known that polymyxin B and colistin have many other antibiotics with which they are able to synergize and create better efficacy or reduce side effects [40,41,42].

Deconinck et al.’s retrospective study [30] (Table 2 and Table 3) showed the differential clinical outcomes from combination therapies and monotherapies. Overall, 134 patients were screened and only 100 patients were included in the study. Of these, 15 received monotherapy while 85 received combination therapy. The isolates in this study, of which 31 were MDR *P. aeruginosa*, were found to be resistant to ceftazidime (31%), piperacillin–tazobactam (34%), cefepime (26%), imipenem (31%), fluoroquinolones (47%), aminoglycosides (45%), and a minority were resistant to colistin (1%). The mortality results after 30 days or discharge consisted of 7 patients dying while on monotherapy (46.7% of the sample size) and 32 patients dying while on combination therapy (37.6%). Deconinck et al. were able to show an indication that combination therapies are effective when compared to monotherapies. This is an overall difference of 9.1% mortality in favour of combination therapies, these results also come from the large study population of 100 patients.

Khawcharoenporn et al. [31] investigated the comparison of monotherapies with two-drug combination therapies (Table 2 and Table 3). This was done by recording the various therapies and the outcomes of those patients who were suffering from XDR *P. aeruginosa* pneumonia at Thammasat University Hospital in Thailand between January 2011 and December 2016. They described the use of antibiotics in patients with susceptible bacteria as “active” and the treatment of patients with resistant organisms as “inactive”. They measured the 14-day survival and microbiological cure rates, as well as the mean survival time and adverse reactions, as gleaned from the physicians’ notes. These factors were compared between the active monotherapies, active combination therapies, and inactive therapies. Overall, it was found that the active therapies resulted in 38 deaths (a 51% mortality rate) while the inactive therapies showed no clinical benefit with 22 deaths (a 100% mortality rate); in contrast, the active combination therapies in this study had a lower, 10% mortality rate with only 4 deaths. This result in turn correlated with the theoretical benefits that combinations have. The mortality increase was found to be correlated with the number of active antimicrobials in the treatments and is reflected in the results for microbiological cure. It was then deemed that synergistic effects were particularly seen between colistin, fosfomycin, and doripenem. This study shows the potential benefits of combination therapy and does so on a comparatively large sample size. Here, direct comparison can be made between the treatments, all of which were treating XDR profile *P. aeruginosa* and, as such, it can be seen that the susceptible combination therapies showed a substantial benefit over the resistant therapies and even the susceptible monotherapies. The weakness of this study is that the sample size was small and that it is retrospective in nature.

In the retrospective observational study that was reported by Kim et al. [32] at Seoul St. Mary’s Hospital, patients who were under the age of 19 and had been diagnosed with *P. aeruginosa* (Table 2 and Table 3) while having hematologic or oncologic comorbidities were observed. It was found that 31 of the patients had episodes of *P. aeruginosa* with 36 total instances of this infection. The cohort had a mean age of 9.5 ± 5.4 and their *P. aeruginosa* infections were found to be moderately resistant to piperacillin–tazobactam and cefepime with only 67.6% and 88.9% susceptibility, which was compared to these isolates being highly susceptible to amikacin, colistin, and ciprofloxacin (100%, 100%, and 97.2%). The patients who did receive a combination therapy were treated with either piperacillin–tazobactam plus aminoglycoside (16 (44.4%)), cefepime with aminoglycoside (2 (5.6%)), or meropenem with aminoglycoside (1 (2.8%)). The monotherapy treatments consisted of either meropenem (14 (38.9%)) or cefepime (3 (7.4%)). Overall, it was found that the combination therapies, as a whole, showed a mortality of 4 (a rate of 21%) while the monotherapies had a mortality of 17 (a rate of 58.8%). The best-performing treatments were cefepime or cefepime with aminoglycoside, both of which had a 0% mortality rate. The sample size was too small for the results to be statistically significant.

Bassetti et al. [33] conducted a phase 3 randomised clinical trial testing the efficacy and safety of cefiderocol as a monotherapy and the best available combination therapies (Table 2 and Table 3). The study was split up such that, out of the total 118 patients who were included in the study, 80 of them received the monotherapy and 38 received the best available combination therapy. Regarding only the *P. aeruginosa* patients, the mortality rates were nearly identical to the mortality rates of the cefiderocol groups (2/12,17%) and the best available therapy groups (2/12, 17%). These mortality rates were assessed on the basis of “all causes” and so they were not specific to the given bacterial infection [33]. The results here indicate there is no difference in mortality, but with the small sample size this should be considered statistically insignificant, therefore it is only indicative of the tested combination therapies providing negligible benefits.

A study (Table 2 and Table 3) that was conducted in the Saudi Tertiary Care Centre by Bosaeed et al. [34] found that, in their study of 19 patients (all of whom were confirmed to have a *P. aeruginosa* infection), every isolate showed resistance to meropenem and imipenem; as such these isolates can be classified as being carbapenem-resistant. Among these isolates, 8 showed susceptibility to at least one aminoglycoside and 10 of the samples were susceptible to cefepime or ceftazidime. However, the majority of the samples (89%) were completely non-susceptible to piperacillin–tazobactam and ciprofloxacin. In the study, 11 of the patients were treated with standard ceftolozane–tazobactam therapy, while the remaining 8 patients were treated with antipseudomonal agents in combination with colistin, aztreonam, and amikacin. At the end of the study, it was found that there was a 21% (4/19) mortality rate in the ceftolozane–tazobactam group after 30 days, but only 2 of those fatalities could be linked to the primary infection; the remaining 2 died due to “complications of catastrophic antiphospholipid syndrome and severe aspiration”. Overall, it can be seen that ceftolozane–tazobactam was an effective choice in the treatments, only resulting in 21% mortality, and considering that 2 (10.5%) of the patients died of further complications it reflects favourably on the effectiveness of the treatment. In this study, there was no comparison with other combinations or monotherapies and so this area cannot be commented on. It can be seen, however, that ceftolozane–tazobactam proved effective against carbapenem-resistant *P. aeruginosa* infections.

A retrospective cohort study by Falagas et al. [35] consisted of 258 patients who were being treated at the Henry Dunant Hospital in Greece (Table 2 and Table 4). This study tested colistin as a monotherapy (*n* = 12), colistin with meropenem (*n* = 28), colistin with piperacillin–tazobactam (*n* = 10), colistin with ampicillin–sulbactam (*n* = 1) and colistin with other agents (*n* = 17). The other agents that were used consisted of aminoglycosides, imipenem, cephalosporins, aztreonam, and ciprofloxacin. The final results were measured by the numbers of clinical cure and deterioration, which are comparable to other mortality data. Colistin monotherapy resulted in 25% (*n* = 3) deterioration, colistin–meropenem therapy resulted in 14.3% (*n* = 4) deterioration, colistin with piperacillin–tazobactam therapy resulted in 40% (*n* = 4) deterioration, colistin with ampicillin–sulbactam therapy resulted in no patient deterioration, and therapy that utilised colistin with other agents resulted in a total of 25.3% (*n* = 6) patient deterioration. Overall, it can be seen that the combination therapies resulted in a 25% rate of patient deterioration (14/56). This study indicates that colistin combinations will match or lower mortality rates, with the exception of piperacillin–tazobactam therapy. This study is difficult to interpret because the numbers are too low for statistical significance and it is not clear which patients died due to resistant or susceptible strains, a factor which is crucial in evaluating whether the treatment can be deemed effective or not. As it stands with the mixed cohort of resistant and susceptible strains in each treatment bracket it is unclear what conclusions can be drawn without these data.

Paul et al. [36] conducted an investigator-initiated, multicentre, open-label, parallel-group, randomised clinical trial between October 2013 and December 2016 (Table 2 and Table 3). Within this study, there were 406 patients who were studied and, of those, only 19 patients had *P. aeruginosa* infections. These patients were split between a colistin monotherapy (*n* = 13) and a colistin–meropenem combination therapy (*n* = 8) condition. By measuring 28-day mortality, the results show that 4 (31%) of the monotherapy patients died and 2 (25%) of the combination therapy patients died over the trial’s duration. Overall, the study showed slight favour towards the colistin–meropenem combination therapy but the sample size being small means that these are statistically insignificant results. It does, however, imply there is some degree of benefit to be had from colistin combinations in the reduction of the potential nephrotoxicity that would usually be caused by colistin use.

Furtado et al. [37] aimed to investigate the effect of polymyxin B treatment on patients with nosocomial pneumonia that was caused by MDR *P. aeruginosa* (Table 2 and Table 3). They conducted a single-centre study at São Paulo Hospital in Brazil, wherein they observed 74 patients. A comparison was drawn between polymyxin B mono- and combination therapies; the combination therapy that was used consisted of imipenem, ciprofloxacin, cefepime, or ceftazidime along with polymyxin B. The majority of the isolates that were tested (*n* = 61, 82.4%) were found to be susceptible to polymyxin B, but were resistant to all of the other antipseudomonal drugs that were used in the study. At the end of the study, it was found that both the combination therapy and the monotherapy resulted in virtually identical clinical effectiveness, with 14 (50%) and 25 (54%) patients having experienced unfavourable outcomes, respectively. This polymyxin B study indicates that there was no discernible difference between the two therapies; there was a marginal reduction in the mortality that was experienced with combination therapies, but this benefit is negated by the smaller sample size. However, it can be seen that all of the isolates that were tested were not susceptible to the other drugs in the combination therapy, indeed this could be looked at as a factor which supports the combination therapy when considering that they didn’t find any nephrotoxicity and reducing a negative side effect such as this would be beneficial.

**Table 4:** Study findings with unclear distribution of resistant and susceptible strains.

Study No.	Treatment	Number of Patients Treated [*n* (%)]	Number of Patients with *P. aeruginosa*		Resistance	Microbiological Cure [*n* (%)]	Mortality [*n* (%)]	References
			Sensitive Strains [*n* (%)]	Resistant Strains [*n* (%)]				
8	Colistin monotherapy	12 (17.6)	135 (52.3) **	123 (47.7) **	MDR	-	3 (25.0) *	[35]
	Colistin–meropenem	28 (41.2)				-	4 (14.3) *	
	Colistin–piperacillin–tazobactam	10 (14.7)				-	4 (40) *	
	Colistin–ampicillin–sulbactam	1 (1.5)				-	0 (0) *	
	Colistin with other agents ^I^	17 (25)				-	6 (25.3) *	
11	β-lactam with aminoglycoside–fluoroquinolone	28 (31.5)	75 (77.3) ^2^	22 (23) ^3^	XDR	-	7 (25)	[38]
	Colistin with other agents	7 (7.9)				-	4 (57.1)	
	β-lactam with β-lactam	2 (2.2)				-	1 (50)	
	β-lactam	26				-	10 (38.5)	
	Colistin	8				-	3 (37.5)	
	Fluoroquinolone	11				-	1 (9.1)	
12	Colistin	4 (14.7)	14 (44.8)	18 (56.2) ^1^	MDR/XDR	6 (35.3)	-	[39]
	β-lactam	15 (44.1)					-	
	Colistin with β-lactam	13 (38.2)				11 (64.7)	-	
	Amikacin with β-lactam	2 (5.9)					-	

* Study used deterioration or “unfavourable outcome” instead of mortality data, exact mortality cannot be ascertained from the study; ** total population values; ^1^ resistance to one drug within treatment; ^2^ resistance to more than one drug within treatment; ^3^ resistant to all drugs within treatment; and ^I^ aminoglycosides (11), imipenem (10), cephalosporins (7), aztreonam (2), and ciprofloxacin (1).

In Greece, Samonis et al. [38] conducted a single-centre retrospective cohort study at the University Hospital of Heraklion (Table 2 and Table 4). Here, 119 isolates were cultured from 109 patients over the course of 7 years. This study focused entirely on patients with cancer. There was a total of 22 cultures that were removed from the analysis for various reasons, this left 97 cultures across 89 patients in the study. Within this study, 6 different therapies were used: 3 combination therapies: β-lactam with aminoglycoside–fluoroquinolone, colistin with “other”, and a double β-lactam therapy; and 3 monotherapies: β-lactam, colistin, and fluoroquinolone. Overall, it was found that the combination therapies resulted in 12 (32.4%) patient deaths, while monotherapy was found to result in 13 (31.1%) patient deaths. This was a cohort wherein it was found that 22 episodes were XDR with resistance to cephalosporins, fluoroquinolones, aztreonam, aminoglycosides, piperacillin–tazobactam, and carbapenems; of the remaining 75 episodes, 5 were MDR and 1 was pandrug-resistant. The small numbers in each cohort makes interpretation difficult.

Ribera et al. [39] conducted a single-centre retrospective cohort study with 34 patients, which focused on osteoarticular infection that was caused by MDR *P. aeruginosa* (Table 2 and Table 4). In this study it was found that out of the 34 patients who were studied, 18 (56.2%) of them had infections which were not susceptible to aztreonam, carbapenems, cephalosporins, and piperacillin–tazobactam. The remainder were at least susceptible to cephalosporins, piperacillin–tazobactam, and carbapenems. This study used clinical cure as a data point, as opposed to mortality. In the end, it was found that, overall, combination therapy had a 64.7% (*n* = 11) clinical cure rate while the monotherapies only had a 35.3% (*n* = 6) clinical cure rate. Like with some other studies, there is no clear way in which to determine which treatments or patients had resistant or susceptible strains. This makes it difficult to conclude the effects that combination therapies might have had on resistant strains. The combination therapies displayed a greater cure rate (64.7%) when compared to the monotherapies (35.3%), but whether or not carbapenem resistance played a role is unclear.

Regarding the efficacy of another combination, imipenem–cilastatin, it has been determined that cefiderocol monotherapy had an absolute difference of 18.58% when compared [43], meaning that in this clinical trial cefiderocol proved to be more effective at treating patients with a wide range of bacterial infections than imipenem–cilastatin. However, in this trial, in order to try to standardise the patients, an initial high-dose treatment of imipenem was used in order to reduce the involvement of *P. aeruginosa* in the test. This led to the patients who continued to have *P. aeruginosa* infections remaining in the test proceedings and showing favourable results from imipenem–cilastatin treatment [43]; these data might suggest that the remaining resistant *P. aeruginosa* responded more favourably to imipenem–cilastatin. It was then demonstrated that cases of complicated urinary tract infections, as well as those patients who were suffering from further complications or other comorbidities, showed a comparable response to imipenem–cilastatin as they did to ceftazidime–avibactam [44]. The finding that these two regimens are largely effective against cases of highly resistant *P. aeruginosa* has been observed previously in this review.

Ceftazidime–avibactam has been shown (Table 5 and Table 6) to be broadly effective for Gram-negative infections [45,46,47,48,49,50]. Its average microbiological cure rate is 65.2% and it has an average clinical cure rate of 73.2% when treating *P. aeruginosa.* In most instances the ceftazidime–avibactam combination performed as well as the comparative treatment, although small differences of <10% in the clinical and microbiological cure rates were observed, with the exception of the results of Mendes et al. [48] who found a 24.5% difference in microbiological cure in favour of ceftazidime–avibactam; however, with regards to clinical cure, there was a 13.4% difference in favour of the best available treatment. Trials which were performed by Qin et al., Wagenlehner et al. and Mazuski et al. [45,46,47] displayed the difference between ceftazidime-susceptible and resistant strains, it can be seen that, in all of these studies, there is a peak difference of 10% clinical cure. Overall, the studies have shown that there is no statistically significant difference between ceftazidime–avibactam and other leading treatments, nor was there a significant difference between the ceftazidime-susceptible and resistant strains.

Throughout this systematic review, some of the data suggest that the use of combination therapies may benefit patients with highly multi-drug resistant and susceptible *P. aeruginosa*.

This evidence has been produced from various countries across many years of relevant study and it includes a wide range of both combination therapies and monotherapies utilising varying drug types [28,29,30,31,32,33,34,35,36,37,38,39,45,46,47,48,49,50]. It can be said that in general the data were insufficient in their quality and the number of patients who were included in order to come to a statistically confirmed conclusion about the advantages or disadvantages of combination therapies versus monotherapy. However, combination therapies seemed to produce a better effect with regard to inhibiting or killing *P. aeruginosa* or an equal effect when compared to the next best available monotherapy.

Ceftazidime–avibactam has been shown to be active against all of the strains of *P. aeruginosa* that were included in one study [51]; however, another found it to be mostly effective and was only found to be non-susceptible to ceftazidime-non-susceptible and extensively drug-resistant (XDR) strains [52,53]. Strains that were found to be hyperproducers of AmpC β-lactamase (5/7) have shown an MIC50 and MIC90 of 64 μg/mL and >64 μg/mL, respectively, when using monotherapy ceftazidime; in comparison, when treated using ceftazidime–avibactam these same strains were seen to have an MIC50 and MIC90 of 8 μg/mL and 32 μg/mL, respectively, despite the populations showing high-class B and D enzymes which would usually lead to high ceftazidime-resistance [54]. This is reinforced by a time kill study which was undertaken in 2021 [55] in which it was found that some XDR strains display up to 128 mg/L MIC when using amikacin which would certainly be determined as resistant; whereas ceftazidime–avibactam saw dramatically lower MICs ranging up to 32 mg/L with a mode of 4 mg/L.

In a clinical setting, it has been found that ceftazidime–avibactam has reportedly showed noticeably lower mortality rates in those who are infected with a carbapenem-resistant *P. aeruginosa* infection when compared to those who are treated with a colistin-based regimen [56]. Supporting this finding, one study reports ceftazidime–avibactam producing similar clinical cure rates to meropenem (91.2% vs. 93.4%), as well as stating its high safety and tolerability when in use. However, this study went on to stipulate that ceftazidime–avibactam should only see use in those patients who are infected with highly resistant strains where other treatments would fail [57]. In one clinical trial of 333 patients across 16 countries, all of whom were suffering from complicated urinary tract infections or complicated intra-abdominal infections, ceftazidime–avibactam therapy was compared with the best available carbapenem monotherapy treatment and it was found that the two methods had the same clinical cure rate (91%) but with an 8% difference in patients experiencing adverse side effects (31% vs. 39%, respectively), of which most were GI complications [53].

There are in vitro studies that show that the results of the microbiological and clinical use of ceftazidime–avibactam are effective, the efficacy of this combination has proven to be greater than that of ceftazidime monotherapy; going from the monotherapies null effect (0% susceptibility) to a greatly decreased MIC and 82.1% susceptibility [52]. In a clinical setting, similar findings were found. This therapy was able to produce comparable clinical cure rates to the likes of meropenem and colistin monotherapies, but with a decrease in adverse effects and lower mortality rates [56,57]. The biggest limitation here would be the inability to inhibit metallo-β-lactamases, such as IMP or NDM, this could lead to ineffectiveness against some strains and as such it cannot be a universal treatment [52]. Similarly, ceftolozane–tazobactam presents almost equal clinical and microbiological effectiveness, even against meropenem-resistant isolates [58,59]. Both therapies can be used in combination with metronidazole, which is highly effective in the treatment of intraabdominal infections due to the prevalence of anaerobic bacteria [60]. The key difference is that avibactam is active against KPC carbapenemases and OXA-48 β-lactamases, whereas tazobactam is not [51].

In another in vitro study, Mei et al. studied 16 isolates of XDR *P. aeruginosa* in order to determine the efficacy of ceftazidime–avibactam. The MICs ranged from 1 to 128 mg/L with the median being 8 mg/L. Only 3 strains had a greater MIC than 8 mg/L. They also studied the MIC of monotherapy colistin against these isolates and found that the range was only 0.5 to 4 mg/L, a significant reduction compared to that of ceftazidime–avibactam. These were then tested in combination with each other in order to give an overall high reduction in MIC with the range of 0.012 to 1 mg/L; this resulted in a calculated fractional inhibitory concentration (FIC) range of 0.313 to 1, showing that an improvement was made with the further combination of ceftazidime–avibactam and colistin within a wide variety of highly resistant strains [61]. Similarly, these findings reflect those of an earlier study from 2011 in which the effectiveness of amikacin combinations was tested on XDR *P. aeruginosa* that had reduced susceptibility to polymyxin B. Here it was found that combination therapies resulted in a much lower log_10_ CFU/mL when compared to the baseline readings; however, 10 isolates were shown to have no bactericidal activity with dual combination treatments, so triple-drug combinations were tested and were largely able to show active effects [62].

Colistin has been considered a critical drug in the treatment of *P. aeruginosa*. However, it is not usually considered as a first-line treatment, rather being restricted to MDR *P. aeruginosa* strains which are known to be colistin-susceptible [63].

While powerful as a monotherapy, colistin has been shown to be more effective in combination therapies. For example, colistin plus tobramycin was proven to be significantly more effective at killing *P. aeruginosa* than any tested monotherapy in static biofilm, dynamic biofilm, and even rat lung biofilm [64]. In monotherapy, colistin was shown to produce bactericidal effects after 24 h [64]. It was then also demonstrated in a different study that colistin treatment alone after the initial 24 h period results in the regrowth of *P. aeruginosa*, despite the sample being mostly colistin-susceptible [65]. It was then, therefore, advised that colistin be used as a combination therapy given the substantial synergy that is seen between it and many carbapenems [41,66]. It is suggested in hollow fibre studies that the use of combination therapy when using colistin demonstrates evidence of resistance suppression [40,67], thereby increasing our chances of an effective treatment for now and in the future. One could argue, however, that this isn’t necessarily indicative of clinical scenarios; but it is near impossible to test clinically as the design of the trial would need to be ethical, which would be hard to achieve in this instance.

The most common adverse side effect of colistin use has been found to be nephrotoxicity, occurring between 10–20% of cases. Comorbidities like chronic renal insufficiency and diabetes mellitus pose an increased chance of nephrotoxicity [68]. However, the pairing of colistin with an aminoglycoside can also increase the risk of nephrotoxicity and would not necessarily yield ideal results when treating osteomyelitis [69]. There is even a debate over the use of colistin in triple antibiotic therapy in order to overcome its shortcomings [70]. In most clinical settings it can be strongly advised that the use of colistin in combination with another anti-pseudomonal should be considered on a case-by-case basis and, as such, to only combine colistin with an antibiotic that poses a potential improvement in the susceptibility of the strain in question [71].

The rate of mutation for *P. aeruginosa* is generally considered to be high. It can be classed as a “hypermutator”, meaning it displays a largely increased mutation rate when compared to other bacteria and so it holds a greater ability to develop resistances to antibiotics [72]. One study did a mutation accumulation experiment on a few types of *P. aeruginosa* (wild type, mismatch repair deficient). They found that the base pair mutation rate was ~5 × 10^−4^ per genome, per generation [73]. While this was found to be lower than was previously thought, it doesn’t necessarily mean that these are the “hypermutator” strains of *P. aeruginosa.* As demonstrated in an Australian study, around 22% of *P. aeruginosa* isolates were found to be hypermutable; with these strains showing greater multidrug resistance than non-hypermutable isolates (38% vs. 22%) [74].

On the basis of these studies, the question of whether or not we should use combination therapies in clinical practice remains unanswered. An international consensus [26] amongst 15 clinicians voted 14 to 1 in favour of combination therapy’s use when treating carbapenem-resistant *P. aeruginosa*. This consensus also stated that this vote was respective of there being little evidence to support the claim that combinations are more microbiologically or clinically effective. This was further exemplified by the committee’s agreement (11-4) to use polymyxin B and colistin in combination with another non-susceptible antibiotic when treating patients with strains that have no available additional susceptible antibiotic to use in combination with polymyxin B or colistin. This strongly implies that, from a clinician’s perspective, combination therapies are largely supported when treating highly resistant pathogens.

## 3. Materials and Methods

In this paper, we have used an electronic literature search; our primary search engine was Ovid, using the Embase, Ovid Medline, and APA PsycInfo databases. This was done in order to find systematic reviews, clinical trials, and randomised controlled trials that were published between January 2010 and January 2021. This time period was chosen in order to keep the data relevant to more recent bacterial strains and the more recent research into newer therapeutic combinations. The final search was run on 25 May 2021 and all of the articles were retrieved by 28 May 2021.

Using this rubric, the search revealed results that adhered to at least one search term from each column. For example, “*P. aeruginosa* MDR Combination therapy” would be one such term (Table 7).

The initial search results were subject to the following inclusion and exclusion criteria (Table 8).

**Figure 1:**
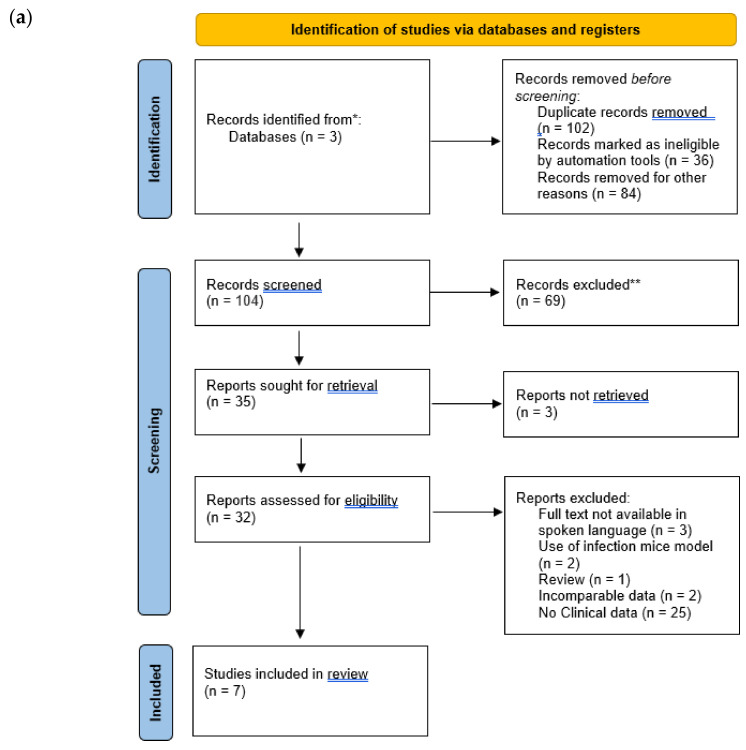

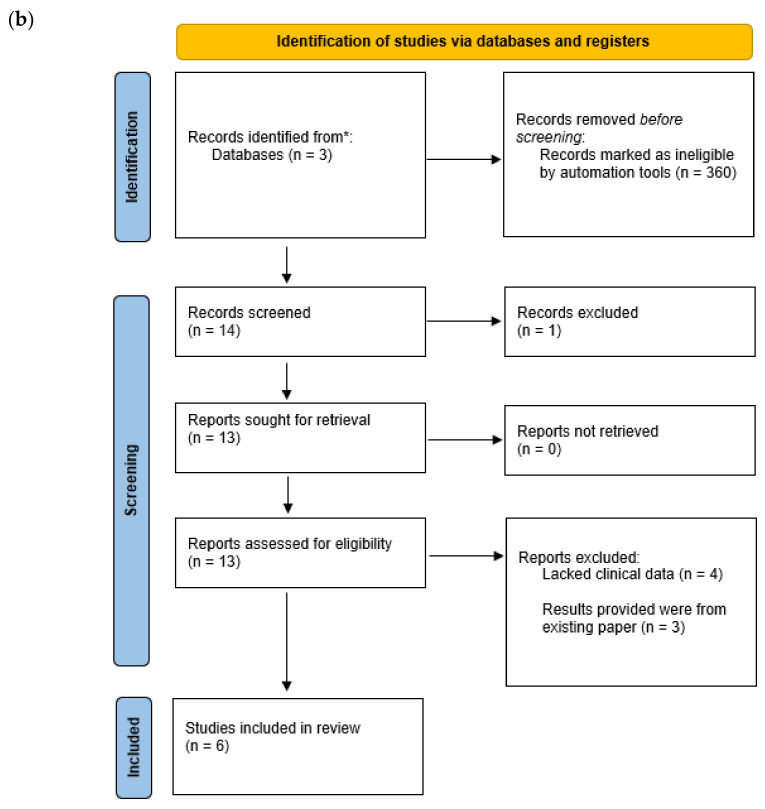
Identification and screening of studies through database and registers. (**a**) Step one of screening. (**b**) step two of screening. * Records identified from databases. ** Records excluded.

The articles that were found went through further screening in order to eliminate irrelevant search results (Figure 1), relevant articles contained mentions of combination therapy drug names and MDR/XDR/Carbapenem-resistant *P. aeruginosa* within their titles or abstracts.

The articles which passed screening were further chosen based on their content consisting of one or more of the following data points: mutation rates of *P. aeruginosa*, combination synergy through fractional inhibitory concentration (FIC) or minimum inhibitory concentration (MIC), hollow fibre data, susceptibility, clinical cure/mortality rates, or market authorisation, specifically by the FDA and EMA. The data that were found were displayed via the use of tables. These tables were then further analysed qualitatively in order to reach a conclusion.

Upon the final selection and screening processes, the articles were assessed for bias. This was solely be based on the acknowledgement of the bias section of each paper (if applicable). If there was found to be a conflict of interest then the paper was not reviewed in this present work.

Two additional papers were used in order to gather more data. One was a review that was undertaken by Samal et al. [75] and the other was the proceedings of a clinicians’ conference on polymyxin use [26]. These articles were searched for their references and these went through the same screening process that was outlined above. Overall, this resulted in an additional 5 studies being included in this review.

A supplementary search was conducted for ceftazidime–avibactam phase 3 clinical trials, this consisted of the same inclusion and exclusion criteria as outlined previously without specifying the inclusion criterion, “articles that are primarily discussions over the topic of combination therapy and *P. aeruginosa*”, as in these papers *P. aeruginosa* is not the focal point of many trials.

## 4. Conclusions

*P. aeruginosa* can rapidly develop resistance, through its many resistance mechanisms, to otherwise highly effective drugs. Combination therapy is widely used for the treatment of resistant *P. aeruginosa*. Whilst the preference of clinicians often favours the use of combination therapies, the over-riding problem with the clinical data for these is that their quality is mostly poor and inconclusive (with some exceptions, such as some ceftazidime–avibactam trials). The high cost of large clinical trials and the relatively low numbers of patients who are infected with *P. aeruginosa* mean that high quality clinical trials are not going to appear in the near future. Clinicians will continue, in the absence of large randomised clinical trials, to use clinical judgement, in vitro data, and the available clinical trials in order to decide whether or not to use combination therapies. This review has assessed the clinical trial data for many of the combinations against resistant *P. aeruginosa.* The efficacy of the combinations that were tested, albeit on the basis of mostly poor clinical trial data, was, on the whole, greater than those of the monotherapies. The combinations demonstrated a reduction in side effects, lower mortality rate, reduced in vitro MIC values, or resistance emergence occurring in favour of the combinations. It was also observed that the treatment needed to have one susceptible antibiotic for it to be successful. This means that infections with bacteria that are resistant to each drug in the regimen were not likely to have a good outcome.

In the future, more clinical trials should be performed with combinations which include colistin and polymyxin B. Although colistin is an old drug, it is still widely used in many countries, it performs at least as well as many other drugs, and it is often used in combinations in which, at least in vitro, it has been shown to be synergistic. The nephrotoxic side effects of colistin seem to be reduced when it is used in combinations. The use of combinations as a whole shows a decrease in MIC when compared to monotreatments and, as such, this suggests that a lower dose could be used in order to reduce any adverse effects.

One of the key in vitro attributes that combination therapy holds is its reduction of the emergence of resistance. This feature can be demonstrated with the hollow fibre infection model. This model has been widely used and can be helpful to clinicians who are deciding which treatment to give to a patient. The likelihood of a randomised clinical trial of sufficient size and quality to be able to address the emergence of resistance during a treatment for *P. aeruginosa* may not be completed for some time.

Although in vitro MIC, synergy, and hollow fibre data predict the clear superiority of some combinations over most monotherapies for resistant *P. aeruginosa*, the clinical trial data do not show a clear advantage, in particular for mortality. This discrepancy is also the case in other Gram-negative infections. Curiously, the in vitro advantages of combinations over monotherapies accurately predict the same advantages for other bacterial diseases such as tuberculosis. This suggests that, for example, the underlying cause of mortality in *P. aeruginosa* infections may be different to that in the case of tuberculosis. In tuberculosis, the cause of death may be due to the direct bacterial infiltration of the heart or a massive bacterial load in the lungs leading to respiratory collapse [76]. In Gram-negative infections, the cause of death is more often multi-organ failure due to a cytokine storm that is not amenable to antibiotic treatment.

In closing, this systematic review indicates that combination therapy is a valid and important treatment option when it comes to treating highly resistant *P. aeruginosa*.

## Figures and Tables

**Table 1:** Current and emerging treatments for *P. aeruginosa* infections.

Mechanism of Resistance	Current Treatments	Emerging Treatments
β-lactamase production	Colistin	Ceftolozane–Tazobactam
Porin loss/mutation	Colistin	Ceftazidime–Avibactam
Efflux pump expression	Colistin	Aztreonam–Avibactam
		Fosfomycin
		Cefiderocol

**Table 2:** Study characteristics included in analysis.

Study No.	Study Title	Study Design		Number of Patients	Clinical Indication	Age		Male Sex *n* (%)	References
						Mean (SD)	Median (IQR)		
1	Epidemiology and outcome of infections with carbapenem-resistant Gram-negative bacteria treated with polymyxin B-based combination therapy	Single	OS	104	Various	77 (±12.9)	-	62 (59.6)	[28]
2	Polymyxin B in Combination with Antimicrobials Lacking In Vitro Activity versus Polymyxin B in Monotherapy in Critically Ill Patients with *Acinetobacter baumannii* or *Pseudomonas aeruginosa* Infections	Multi	RS	101	Various	65.2 (±15.7)	-	56 (55.4)	[29]
3	Impact of combination therapy and early de-escalation on outcome of ventilator-associated pneumonia caused by *Pseudomonas aeruginosa*	Single	RS	100	VAP	-	64 (54–72)	76 (76.0)	[30]
4	Active monotherapy and combination therapy for extensively drug-resistant *Pseudomonas aeruginosa* pneumonia	Single	RS	136	Various	-	78 (70–83)	74 (54)	[31]
5	Clinical characteristics and outcomes of *Pseudomonas aeruginosa* bacteremia in febrile neutropenic children and adolescents with the impact of antibiotic resistance: a retrospective study	Single	ROS	31	Febrile neutropenia	9.5 (±5.4)	-	26 (72.2)	[32]
6	Efficacy and safety of cefiderocol or best available therapy for the treatment of serious infections caused by carbapenem-resistant Gram-negative bacteria (CREDIBLE-CR): a randomised, open-label, multicentre, pathogen-focused, descriptive, phase 3 trial	Multi	RCT	118	Various	63.0 (±16.7)	-	101 (66.4)	[33]
7	Experience with Ceftolozane–Tazobactam for the Treatment of Serious *Pseudomonas aeruginosa* Infections in Saudi Tertiary Care Center	Single	ROS	19	Various	-	57 (35–71)	9 (47)	[34]
8	Colistin therapy for microbiologically documented multidrug-resistant Gram-negative bacterial infections: a retrospective cohort study of 258 patients	Single	RS	258	Various	61.1 (±18.1)	-	174 (67.4)	[35]
9	Colistin alone versus colistin plus meropenem for treatment of severe infections caused by carbapenem-resistant Gram-negative bacteria: an open-label, randomised controlled trial	Multi	RCT	406	Various	66 (±16)	-	151 (37.2)	[36]
10	Intravenous polymyxin B for the treatment of nosocomial pneumonia caused by multidrug-resistant *Pseudomonas aeruginosa*	Single	OS	74	Nosocomial pneumonia	-	55 (17–89)	50 (67.6)	[37]
11	Characteristics, risk factors and outcomes of adult cancer patients with extensively drug-resistant *Pseudomonas aeruginosa* infections	Single	RS	89	Cancer	-	73 (21–87)	18 (81.8)	[38]
12	Osteoarticular infection caused by MDR *Pseudomonas aeruginosa*: the benefits of combination therapy with colistin plus β-lactams	Single	RS	34	Osteoarticular infection	-	69 (60–78)	20 (58.8)	[39]

Single refers to single-centre; multi refers to multicentre; OS, observational study; RS, retrospective cohort study; RCT, randomised clinical trial; ROS, retrospective observational study; POS, prospective observational cohort study; SD, standard deviation; and IQR, interquartile range.

**Table 3:** Study findings with results separated by treatment.

Study No.	Treatment	Number of Patients Treated [*n* (%)]	Number of Patients with *P. aeruginosa*		Resistance	Microbiological Cure [*n* (%)]	Mortality [*n* (%)]	References
			Sensitive Strains [*n* (%)]	Resistant Strains [*n* (%)]				
1	Polymyxin B (combination) ^a^	104	0 (0)	11 (10.5)	Carbapenem resistant	20	50	[28]
2	Polymyxin B (combination) ^b^	33 (34.7)	0 (0)	3 (9.1) ^1,^**	XDR	-	0 (0)	[29]
	Polymyxin B (monotherapy) ^c^	68 (65.3)	15 (22) **	0 (0)	XDR			
3	Empirical combination therapy ^c^	85 (85.0)	0 (0)	26 (31) ^2^	MDR	-	32 (37.6)	[30]
	Empirical monotherapy ^d^	15 (15)	0 (0)	5 (33) ^2^	MDR	-	7 (46.7)	
4	Susceptible combination ^e^	40 (29.4)	40 (29.4) ^2^	0 (0)	XDR	36 (90)	4 (10)	[31]
	Susceptible monotherapy ^f^	74 (54.4)	74 (54.4) ^2^	0 (0)	XDR	40 (54)	38 (51)	
	Resistant therapy ^g^	22 (16.2)	0 (0)	22 (16.2) ^3^	XDR	0 (0)	22 (100)	
5	Piperacillin–tazobactam with aminoglycoside	16 (44.4)	0 (0)	16 (100) ^1^	MDR	-	3 (21.4)	[32]
	Meropenem	14 (38.9)	14 (100)	0 (0)	MDR	-	10 (71.4)	
	Cefepime	3 (7.4)	3 (100)	0 (0)	MDR	-	0 (0)	
	Cefepime with aminoglycoside	2 (5.6)	2 (100)	0 (0)	MDR	-	0 (0)	
	Meropenem with aminoglycoside	1 (2.8)	1 (100)	0 (0)	MDR	-	1 (100)	
6	Cefiderocol	80 (67.8)	12 (15)	0 (0)	Carbapenem resistant	-	2 (17)	[33]
	Cefiderocol combination ^h^	38 (32.2)	10 (26)	0 (0)	Carbapenem resistant	-	2 (20)	
7	Ceftolozane–tazobactam	19 (100)	19 (100)	0 (0)	Carbapenem resistant	14 (74)	4 (21)	[34]
9	Colistin	198 (48.8)	13 (4)	0 (0)	Carbapenem resistant	-	4 (31)	[36]
	Colistin–meropenem	208 (51.2)	0 (0)	8 (3.8) ^1^	Carbapenem resistant	-	2 (25)	
10	Polymyxin B	46 (62.2)	46 (62.2)	0 (0) **	MDR	-	25 (53) *	[37]
	Polymyxin B combination ^i^	28 (37.8)	0 (0)	28 (37.8) ^2,^**	MDR	-	14 (50) *	

* Study used deterioration or “unfavourable outcome” instead of mortality data, exact mortality cannot be ascertained from the study; ** susceptible to polymyxin B; ^1^ resistance to one drug within treatment; ^2^ resistance to more than one drug within treatment; ^3^ resistant to all drugs within treatment; ^a^ polymyxin B with carbapenem, polymyxin B with carbapenem and rifampin, polymyxin B with ampicillin–sulbactam, or polymyxin B with carbapenem and tigecycline; ^b^ polymyxin B with “an antimicrobial lacking in vitro activity”; ^c^ β-lactam–aminoglycoside 85 (85%), β-lactam–fluoroquinolone 20 (23.5%), β-lactam–aminoglycoside–fluoroquinolone 2 (2.3%), fluoroquinolone–aminoglycoside 1 (1.2%), combination with colistin 9 (10.6%); ^d^ β-lactam 9 (60%), aminoglycoside 3 (20%), fluoroquinolone 2 (13.3%), colistin 1 (6.7%); ^e^ colistin with fosfomycin (*n* = 22; 55%), doripenem with fosfomycin (*n* = 12; 30%), colistin with doripenem (*n* = 6; 15%); ^f^ colistin and non-active carbapenem (*n* = 40; 54%), colistin alone (*n* = 22; 30%), colistin and non-active fosfomycin (*n* = 6; 8%), fosfomycin and non-active carbapenem (*n* = 4; 5%), doripenem and non-active fosfomycin (*n* = 2; 3%); ^g^ piperacillin–tazobactam (*n* = 10; 46%), non-active carbapenems (*n* = 10; 46%), non-active fosfomycin and non-active carbapenems (*n* = 2; 9%); ^h^ cefiderocol treatment combined with one adjunctive antibiotic, excluding polymyxins, cephalosporins (including β-lactamase inhibitor combinations), and carbapenems; and ^i^ aminoglycosides (11), imipenem (10), cephalosporins (7), aztreonam (2) and ciprofloxacin (1).

**Table 5:** Study characteristics included in ceftazidime–avibactam analysis.

Study No.	Study Title	Study Design		Number of Patients	Clinical Indication	Age		Male Sex *n* (%)	Reference
						Mean (SD)	Median (IQR)		
13	A randomised, double-blind, phase 3 study comparing the efficacy and safety of ceftazidime–avibactam plus metronidazole versus meropenem for complicated intra-abdominal infections in hospitalised adults in Asia	Multi	RCT	431	cIAI	48.5 ± 16.8	-	294 (68.2)	[45]
14	Ceftazidime–avibactam Versus Doripenem for the Treatment of Complicated Urinary Tract Infections, Including Acute Pyelonephritis: RECAPTURE, a Phase 3 Randomized Trial Program	Multi	RCT	810	cUTI	51.4 ± 20.2	-	245 (30.2)	[46]
15	Efficacy and Safety of Ceftazidime–Avibactam Plus Metronidazole Versus Meropenem in the Treatment of Complicated Intra-abdominal Infection: Results from a Randomized, Controlled, Double-Blind, Phase 3 Program	Multi	RCT	1043	cIAI	49.8 ± 17.5	-	658 (63.1)	[47]
16	Characterization of β-Lactamase Content of Ceftazidime-Resistant Pathogens Recovered during the Pathogen-Directed Phase 3 REPRISE Trial for Ceftazidime–Avibactam: Correlation of Efficacy against β-Lactamase Producers	Multi	RCT	295	Various	-	-	-	[48]
17	Clinical activity of ceftazidime–avibactam against MDR Enterobacteriaceae and *Pseudomonas aeruginosa*: pooled data from the ceftazidime–avibactam Phase III clinical trial programme	Multi	RCT	1051	Various	-	-	-	[49]
18	Ceftazidime–avibactam versus meropenem in nosocomial pneumonia, including ventilator-associated pneumonia (REPROVE): a randomised, double-blind, phase 3 non-inferiority trial	Multi	CT	726	VAP	-	62·1 (16·6)	542 (74.7)	[50]

Multi refers to multicentre; RCT, randomised clinical trial; CT, clinical trial; cIAI, complicated intra-abdominal infection; cUTI, complicated urinary tract infection; SD, standard deviation; and IQR, interquartile range.

**Table 6:** Study findings from the supplementary ceftazidime–avibactam search.

Study No.	Treatment	Number of Patients with *P. aeruginosa* [*n* (%)]	Ceftazidime Resistance Profile	Number of Patients Treated [*n* (%)]	Microbiological Cure [*n* (%)]	Clinical Cure [*n* (%)]	References
13	Ceftazidime–avibactam	11 (2.6)	Res	1 (9.1)	-	1 (100)	[45]
			Sus	10 (90.9)	-	10 (100)	
14	Doripenem	37 (4.6)	Res	6 (16.2)	5 (83.3)	-	[46]
			Sus	14 (37.8)	10 (71.4)	-	
	Ceftazidime–avibactam		Res	7 (18.9)	5 (71.4)	-	
			Sus	10 (27.0)	7 (70.0)	-	
15	Ceftazidime–avibactam and metronidazole	68 (6.5)	Res	2 (2.9)	-	2 (100)	[47]
			Sus	30 (44.1)	-	27 (90.0)	
	Meropenem		Res	4 (5.9)	-	4 (100)	
			Sus	32 (47.0)	-	30 (93.8)	
16	Ceftazidime–avibactam	18 (6.1)	-	13 (72.2)	11 (84.6)	11 (84.6)	[48]
	Best available treatment		-	5 (27.8)	3 (60.0)	5 (100)	
17	Ceftazidime–avibactam	95 (9.0)	66.1% Sus	56 (58.9)	32 (57.1)	-	[49]
	Carbapenem comparators			39 (41.1)	21 (53.8)	-	
18	Ceftazidime–avibactam	77 (10.6)	24.8% Sus	42 (54.5)	18 (42.9)	27 (64.3)	[50]
	Meropenem			35 (45.5)	14 (40.0)	27 (77.1)	

Res—resistant and sus—susceptible.

**Table 7:** Key words used for the literature search.

Search Term 1	Search Term 2	Search Term 3
*Pseudomonas aeruginosa*	MDR	Combination therapy
	XDR	Multi-drug therapy
	Carbapenem resistant	
	Multi-drug resistant	
	Antibiotic resistant	
	Extensively drug resistant	

**Table 8:** Inclusion and exclusion criteria for the literature search.

Inclusion Criteria	Exclusion Criteria
Title or abstract must include a reference to antibiotic combination therapy or a name of one or more antibiotic combinations	Articles without an available abstract or full text
Title or abstract must include a reference to *P. aeruginosa*	Articles that were not published
Title or abstract must include a reference to antibiotic-resistant strains	Articles indicating that *P. aeruginosa* or antibiotic combination therapy and its efficacy is not the main focus
Must include in vitro or in vivo studies	Reviews, conference abstracts etc.
Papers that include data on MIC or FIC	Articles that are primarily discussions of the topic of combination therapy and *P. aeruginosa*
Papers that include monotherapy data	All surveys
Papers that include clinical cure, mortality rate, or other clinical data	Articles with simulated testing methodologies
Papers that include % susceptibility data	
